# A Review Study on the Trends of Psychological Challenges, Coping Ways, and Public Support During the COVID-19 Pandemic in the Vulnerable Populations in the United States

**DOI:** 10.3389/fpsyt.2022.920581

**Published:** 2022-07-07

**Authors:** Rakesh Kumar, Anand Singh, Rahul Mishra, Ushasi Saraswati, Jaideep Bhalla, Sandeep Pagali

**Affiliations:** ^1^Mayo Clinic, Rochester, MN, United States; ^2^Institute of Human Behaviour and Allied Sciences, University of Delhi, New Delhi, India; ^3^Maulana Azad Medical College, University of Delhi, New Delhi, India

**Keywords:** COVID-19, psychosocial support systems, mental health, psychological adaptation, culture, society

## Abstract

**Background:**

The COVID-19 pandemic resulted in significant mortality and morbidity in the United States. The mental health impact during the pandemic was huge and affected all age groups and population types. We reviewed the existing literature to understand the present trends of psychological challenges and different coping strategies documented across different vulnerable sections of the United States population. This rapid review was carried out to investigate the trends in psychological impacts, coping ways, and public support during the COVID-19 pandemic crisis in the United States.

**Materials and Methods:**

We undertook a rapid review of the literature following the COVID-19 pandemic in the United States. We searched PubMed as it is a widely available database for observational and experimental studies that reported the psychological effects, coping ways, and public support on different age groups and healthcare workers (HCWs) during the COVID-19 pandemic.

**Results:**

We included thirty-five studies in our review and reported data predominantly from the vulnerable United States population. Our review findings indicate that COVID-19 has a considerable impact on the psychological wellbeing of various age groups differently, especially in the elderly population and HCWs. Review findings suggest that factors like children, elderly population, female gender, overconcern about family, fear of getting an infection, personality, low spirituality, and lower resilience levels were at a higher risk of adverse mental health outcomes during this pandemic. Systemic support, higher resilience levels, and adequate knowledge were identified as protecting and preventing factors. There is a paucity of similar studies among the general population, and we restricted our review specifically to vulnerable subgroups of the population. All the included studies in our review investigated and surveyed the psychological impacts, coping skills, and public support system during the COVID-19 pandemic.

**Conclusion:**

The evidence to date suggests that female gender, child and elderly population, and racial factors have been affected by a lack of support for psychological wellbeing. Further, research using our hypothesized framework might help any population group to deal with a pandemic-associated mental health crisis, and in that regard, analysis of wider societal structural factors is recommended.

## Introduction

World Health Organization (WHO) declared the novel coronavirus (2019-nCoV) a public health emergency of international concern (PHEIC) on 30 January 2020, and a pandemic on 11 March 2020 (1). Thereafter, the COVID-19 pandemic has negatively affected the mental health of various population groups. This ongoing undesirable grim has significant mental health implications across all age groups and even for health professionals in the United States, and a mental health crisis has emerged. Initially, the United States Centers for Disease Control and Prevention (CDC) estimated that nearly one-third of the United States adults have anxiety or depression as on June 2020 (2). A major media, Usatoday.com, came up with the headline “Mental illness is epidemic within the coronavirus pandemic,” reflecting the depth of the mental-health crisis in the United States.

There is a dual magnitude of concern uprising mental health (suicidal ideation) and substance use disorders, as well as the onset of new barriers (3, 4). The United States Census Bureau, household pulse survey, reported similar data that 41% of adults reported symptoms of depressive and/or anxiety disorder in January 2021 as compared with January 2019 (5). As the pandemic wears on, necessary and ongoing public health measures exposed many persons to experience situations linked to mental health conditions, such as isolation and job loss. It is very crucial for each country to understand its impact on public health and its effects on the impact of Mental Illness, Culture, and Society. A recent study reported that COVID-19 deaths were underestimated and later on increased the inappropriateness of this pandemic situation in the United States (6). The study projected that COVID-19 had resulted in enormous losses of American lives, and this was highlighted by the national media in Washingtonpost.com, which made headlines stating that *the toll went beyond the number of civil war deaths in past*. The United States government came up with various effective strategies and rigorous attempts for the strict implementation of COVID-19 appropriate behaviors and vaccination, which lead to indispensable public involvement. The massive media coverage increased awareness about the science and pseudoscience of the pandemic combined with uncertainty and evolution of new viral variants that have effectively contributed to the “pandemic fatigue” with the potential of more spread of infection and further taken a toll on mental health issues. In addition, a large community-based study found vaccine hesitancy in 22% of the participants in the United States during the COVID-19 pandemic (7). The current pandemic is significantly impacting the mental health of all over the past 2 years. The vulnerable section of the population (youth, pregnant women, elderly, and healthcare workers) is particularly impacted during pandemics.

In this review study, we aimed to assess the psychological challenges of specific age groups, working groups, and their possible coping mechanisms. In this study, we conducted a narrative review on the trends of psychological challenges, coping ways, and public support for the groups such as young adults, pregnant ladies, healthcare workers (HCWs), and the elderly population.

## Aim of the Review

This review aims to identify the psychological impacts, coping mechanisms, and public support during the COVID-19 pandemic in different population groups (child and adolescent, pregnant and postpartum women, minority racial population, elderly population, and healthcare professionals). The second aim was to identify risks and protective factors associated with adverse mental health outcomes. This rapid review and robust gathering of evidence could be used to inform governments/healthcare decision-makers, which will be vital to future policy making.

## Materials and Methods

### Search Strategy

We planned, conducted, and reported this study according to the guidelines for rapid reviews (8), WHO (9), and the recent Cochrane Collaboration’s recommendations for COVID-19 (10).

### Data Sources and Searches

Two authors (US and RM) searched across PubMed, a widely available database to capture research from potentially relevant fields, including health, mental health, and health management. The search strategy was executed on 14 February 2022, and again 2 weeks later on 28 February 2022, using a combination of subject headings and keyword searching. The bibliographical database was created with EndNote X9™.

### Search Criteria

**FIGURE 1:**
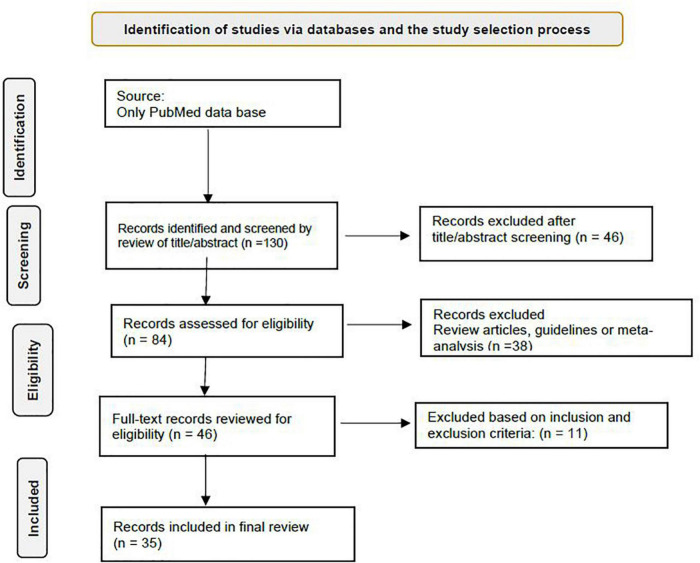
Study flowchart.

The design of the search criteria was intended to draw together research both for this rapid review and to contribute to the design of a digital mental health intervention to enhance the psychological wellbeing of different populations including HCWs. We used keywords such as COVID-19, United States, mental health, psychosocial support systems, and psychological adaptation for the search of studies. The flowchart for the study is presented in Figure 1.

### Types of Participants

Participants were restricted to studies based in the United States on different age groups, races, pregnancy-related populations, and HCWs during the COVID-19 pandemic. The findings in each population type, namely, the child, adolescent, and young adult population, elderly adult population, healthcare workers, pregnant and post-partum women, and minority ethnic groups, were studied. Based on these documented observations, we propose a framework for clinicians’ consideration to identify the etiology of mental illness during these times and accordingly consider the coping strategies.

### Types of Studies Included

Published observational and experimental survey studies that reported the psychological effects, coping skills, and public support during the COVID-19 pandemic were included. The study designs included quantitative and qualitative primary studies. Studies relating to any previous pandemics and other epidemics (such as H1N1, H5N1, SARS, MERS, Zika, Ebola, and West Nile Fever) were excluded. All studies other than United States and published in languages other than English were excluded.

### Screening and Selection of Studies

Searches were screened according to the selection criteria by RM. The full text of potentially relevant studies/papers was retrieved for further clarification. Data extraction and quality assessment were done by RM, US, and JB. Relevant data were extracted into structured tables including study, population, age, and psychological symptoms. Common coping methods and main study results were reported, wherever available, and authors extracted protective factors and risk factors.

Tables 1, 2 present an overview of the included studies. RK and SP assessed the quality of studies and assessed their risk of bias using the Evidence Partners appraisal tool. Data synthesis and analysis were conducted. The outcomes were categorized according to the study, population, age, and psychological symptoms/impact of COVID-19.

**TABLE 1:** An overview of the included studies on child and adolescent, and HCWs studies.

Study	Mode	Population (n)	Age (Mean SD)	Psychological symptoms	Common coping methods reported
Garritty et al. (10)	Survey	195	21 (1.7)	Fear/worry about health (91%); sleep pattern disruption (86%); difficulty in concentration (89%); increased concerns about academic performance (82%)	Negative coping (ignoring COVID-19 news, sleeping longer, distraction, drinking, and smoking), positive coping (meditation and breathing exercises, spiritual measures, keeping routines, and positive reframing), self-management
Son et al. (11)	Survey	2,031	23 (5.5)	Increase in preexisting anxiety (71%), depression (48%), anxiety (38%), and suicidal thoughts (18%)	Support from others, smartphone apps, university health services
Wang et al. (12)	Survey	1,015	39 (13.5)	Variable stressors related to COVID-19 infection	Distraction, active coping, and seeking emotional social support
Rosen et al. (15)	Survey	484	18 (0.3)	Mood decline	–
Park et al. (13)	Survey	950	19 (2.8)	Anxiety or depression	Staying connected and maintaining positivity
Hamm et al. (28)	HCW Survey	657	–	Depression (48%), anxiety (33%) and acute stress (48%)	Physical therapy or exercises
Shechter et al. (29)	HCW Survey	288	46 (11.5)	Stress, increased anxiety/depression	–
Comfort et al. (30)	HCW Survey	517	–	Insomnia (18%), depression (17%), anxiety (13%) PTSD (7.5%)	Avoidance coping, humor, positive reframing
Daly and Robinson et al. (46)	Survey	126	73 (7.4)	Depressed mood (27%), loss of interest (21.4%), change in sleep quality (25.1%), and change in alcohol use (6.4%)	Adaptive coping methods

*HCWs, healthcare workers.*

**TABLE 2:** An overview of the included studies on elderly populations, pregnant, and minorities.

Study #	Mode	Population # (n)	Age	Psychological symptoms	Common coping methods reported
LaCaille et al. (18)	Survey	Elderly (6,938)	67.3 ± 7.9	Leaving home only for essentials (69%), Placed on leave of absence/furloughed (in 55–74 years = 17%; in ≥75 years = 31%), Screening positive for: depression (32%), anxiety (29%), loneliness (29%)	NA (only looked at outcomes)
Kobayashi et al. (19)	Survey	Elderly (6,548)	67.7 ± 0.2	Increase in alcohol consumption compared to pre-COVID drinking (11%), Association with increased drinking: depression: OR = 2.66, 95% CI: 1.99–3.56; anxiety: OR = 1.80, 95% CI: 1.34–2.42; loneliness: OR = 2.45, 95% CI: 1.83–3.28. If positive for all 3, more likely to report increased alcohol consumption (OR = 3.87, 95% CI: 2.52–5.96, vs. no mental health outcomes)	Increased alcohol consumption was a coping mechanism for COVID-19 stressors
Eastman et al. (20)	Survey	Elderly (1,714)	Age was reported in 5-year increments from 60–90+, for a total of 7 response options coded 1–7; (mean = 2.35 ± 1.25)	pre-virus annual income was reported in $25k increments from $0 to $150k+, for a total of 7 response options coded 1–7; marital status was coded 1 = single/divorced/widowed, 2 = married/partnered; and retirement status was coded 1 = fully retired, 2 = work part time or full-time. Perceived health was rated on a 4-point scale: 1 = very healthy (39.6%), 2 = somewhat healthy (52.1%), 3 = not very healthy (7%), 4 = in poor health (1.3%). Mean ± SD for variables were Income = 3.78 ± 1.7; Marital status = 1.7 ± 0.46; Retired = 1.34 ± 0.47, Health = 1.7 ± 0.65, Income decline = 151 ± 0.5, Perceived stress = 17.61 ± 2.93, Negative affect = 35.08 ± 4.05	NA
Whitehead (21)	Survey	Adults (515)	39.48 ± 11.85	Both knowledge and precautions remained related to stress and that anxiety about developing COVID-19 contributed a large portion of the variance (β = 0.66) but health was no longer significant. For the Age × COVID-19 Anxiety interaction, anxiety was associated with more COVID-19 stress for older adults relative to younger adults	Knowledge of COVID-19. Knowledge of precautions, proactive coping, education
Pearman et al. (22)	Survey	Elderly (6,938)	67.3 ± 7.9	NA (study was on coping methods used)	exercising and going outdoors (26%), modifying routines (25%), following public health guidelines (18.9%), adjusting attitudes (16.1%), and staying socially connected (15.3%). 20% used no coping methods. Some coping strategies were health-limiting (e.g., overeating) (1.1%)
Finlay et al. (23)	Survey	Elderly (430)	72.4 ± 6.7	Risk perception: Most considered themselves to be high risk due to (a) underlying health conditions and (b) due to age Financial impact: mixed (as retired, mostly) Emotions: anxiety, fear, loneliness, lack of social connections	Coping was problem- and emotion-focused. Problem-focused coping included precautionary efforts and emotion-focused coping included creating daily structure, pursuing new and/or creative activities, connecting with others in new ways, and minimizing news media exposure
Goins et al. (24)	Survey	Elderly (141)	74.36 ± 8.35	Perceived stress via PSS-14 questionnaire = 23.5 ± 5.2 (moderate); inversely related to age (*r* = -0.29, *p* = 0.001); more in women (*t* = 2.05, df = 135, *p* = 0.042); divided into: health of loved ones (most common), self-health, finances, cognition, sleep, appetite	The three most endorsed coping strategies were acceptance (mean and SD = 2.5 ± 0.8), positive reframing (mean and SD = 1.84 ± 1.06) and active coping (mean and SD = 1.7 ± 1.0), and the three least endorsed coping strategies were behavioral disengagement (mean and SD = 0.2 ± 0.5), substance use (mean and SD = 0.3 ± 0.7) and self-blame (mean and SD = 0.5 ± 0.7). The highest endorsed coping strategy was I’ve been eating healthy and well-balanced meals (mean and SD = 2.4 ± 0.8) and the least endorsed coping strategy was I’ve been practicing mindful movements (e.g., Yoga, Qigong and Tai Chi) (mean and SD = 0.7 ± 1.0)
Vannini et al. (25)	Survey	Adults (13,180)	52.42 ± 17.76	Posttraumatic stress was highly correlated with the psychosocial outcome variables of depression, anxiety, and loneliness. Objective social isolation (e.g., having limited contact with family and friends) was related to stress	Avoidant coping, approach coping, social support
Czeisler et al. (34)	Survey	Peripartum (162)	31 ± 4.8	Stress/anxiety; decline in nutrition, missed appointments; access to baby supplies; less in-health facility deliveries. Financial resources, COVID-19 information and research as it relates to maternal-infant health outcomes, access to safe healthcare, and access to baby supplies (formula, diapers, etc.) emerged as the primary resources needed by participants	Support from friends and family, telemedicine, birthing classes, counseling services, better screening for stressors
Barbosa-Leiker et al. (35)	Survey	Peripartum (527)	32.60 ± 4.52	Predictors of depressive symptoms, anxiety, and post-traumatic stress disorder were analyzed The most common predictors were job insecurity, family concerns, eating comfort foods, resilience/adaptability score, sleep, and use of social and news media. Qualitative themes centered on pervasive uncertainty and anxiety; grief about losses; gratitude for shifting priorities; and use of self-care methods including changing media use	Social support (84%) increased social media use (48%), sleep (44%), eating comfort foods (42%), decreasing news intake (42%), exercising (36%), peer support (32%). Harmful = alcohol (10%), other substance use (5%)
Kinser et al. (36)	Survey	Perinatal (60)	32.3 ± 3.8	Over three-fourths of the sample indicated a worsening of mental health during the pandemic, with 31.7% of women endorsing clinically elevated depression symptoms and 36.7% screening positive for anxiety	Domestic tasks, increased time w/baby for postnatal, being in nature, social support, distracting oneself, exercise and healthy behaviors, better food and water habits, extended breastfeeding w/o need of pumping. Some also had avoidant coping mechanisms which were harmful
Anderson et al. (37)	Survey	Latinx, Latin, and Hispanic (341)	40 ± 11.6	Respondents who identified as Latinx, Latina, or Hispanic were 10 times more likely to meet the threshold score for depression (*X*^2^ = 7.21, *p* = 0.007). Similarly, individuals with prior mental health conditions (*X*^2^ = 12.22, *p* = 0.001) and those who expressed feelings of social isolation due to COVID-19 (*X*^2^ = 10.07, *p* = 0.002) were 3 times more likely to meet the threshold score for depression. As age (*r* = −0.25) and income (*r* = −0.20) increased, respondents were less likely to have prior mental health problems. Individuals who reported social isolation lived in communities with higher percentages of people in fair or poor health (*r* = 0.20). and were younger (*r* = −0.25)	NA
Saltzman et al. (38)	Survey	Latina (King County) (137)	42 ± 10.6	Very few women had been infected with COVID-19, and 23% reported having been tested. Most frequent reasons for not being tested were not knowing where to go (14%), concerns over the cost (15%), and not wanting to know if they were infected (12%). Most participants had concerns about paying for housing (76%) and food (73%). Depression and anxiety symptoms were in the moderate range	Recommended preventive behaviors followed. Coping methods not discussed
Ornelas et al. (39)	Survey	Latinx (underserved) (43)	45 ± 11.1	Six themes related to mental health stressors including economics (e.g., job insecurity), immigration (e.g., undocumented status), misinformation, family stress (e.g., changes in family dynamics and the home environment), health (e.g., limited healthcare access) and social isolation	Coping skills of the community were categorized into four themes with multiple codes including behavioral strategies (e.g., identifying reliable information, relaxation, mindfulness, stimulus control), cognitive strategies (e.g., collectivistic thinking, gratefulness, self-compassion), social support and spirituality (faith, religiosity)
Garcini et al. (40)	Perspective study	African American	NA	Closure of African American churches (called as “Black Church”) led to increased mental stress to the followers, as they served as a historical and cultural symbol for them and improved mental health in the community members dealing with racism	NA
DeSouza et al. (41)	Survey	African American adolescents (12)	12–18	Participants struggled with adjusting to the changes in their daily routines, navigating virtual learning, and emerging mental health difficulties (e.g., anxiety)	Participants relied on emotion and problem-focused coping strategies, including strategies that were religious/spiritual in nature. Participants also relied on social support from family, school personnel, and their religious community
Parker et al. (42)	Survey	Korean immigrants (790)	45.74 ± 12.14	In terms of psychological distress, almost half of the sample (49.4%) had a low level of psychological distress. The other half (49.2%) had a high level of psychological distress. A person’s resilience was the most important predictor of the level of respondents’ psychological distress (Importance 0.173; Normalized importance 100.0%), followed by experiences of everyday discrimination (Importance 0.144; Normalized importance 83.2%), COVID-19 discrimination (Importance 0.144; Normalized importance 59.8%) and social support (Importance 0.095; Normalized importance 55.1%)	Resilience, social support
Choi et al. (43)	Survey	American Orthodox Jews (419)	39.17 ± 15.71	Participants reported significantly less than average impact of COVID-19 on religious observance, faith in God, and their character (e.g., patience, trust), and significantly more impact on sleep, fitness, work, family, finances, and emotions. A similar pattern of correlations was observed for secondary exposure via news media, social media, and one-on-one communications, which correlated with higher self-reported negative impact overall	Positive religiosity

*HCWs, healthcare workers.*

## Results

### Study Characteristics

In this review, we included 35 studies, which contain surveys and data from the predominantly United States-based studies.

### Psychological Toll and Coping Ways in Children, Adolescent, and Young Adult Population

Various surveys were conducted to assess the effect of COVID-19 on mental health among college students. In a survey by Son et al. (11), participants reported fear/worry about their health, sleep pattern disruptions, difficulty in concentration, and increased concerns about their academic performance, whereas another survey by the same authors showed increments in preexisting anxiety, depression, anxiety, and suicidal thoughts (12). However, 43% of participants could cope adequately with stress. The restrictions in social interaction, uncertain duration, and severity of illness were perhaps the reasons for these alarming survey results. Another study result showed that reading/hearing about the severity and contagiousness of COVID-19 was reported as the most common stressor (13). Overall, their financial concerns were rated as the most stressful factor. The most common treatments were distraction techniques, active coping, and seeking emotional support for social stress management. In addition, Waselewski et al. observed that 35.2% of the United States youth reported unavailability of appropriate resources during the early phase of COVID-19 (14). This study also concluded that the emotional responses were mostly negative (anxiety or depression), while coping strategies included staying connected and maintaining positivity.

Interestingly, another study by Rosen et al. (15) investigated the association between pandemic-related stressors and psychopathology, which was found to be reduced among youths (*n* = 224, mean age 12.6 ± 2.6) who had limited passive screen time, and it was absent in children having lower news media consumption related to the pandemic, but this was not seen in adolescents age group. Rettew et al. (16) conducted a personality assessment survey with the Big Five Inventory (BFI) among college students to find the association of traits with adjustment to the COVID-19 pandemic. The authors found that a higher proportion of extraversion openness, agreeableness, and conscientiousness and a lower proportion of neuroticism were associated with positive outcomes. However, there was an increased perceived threat and a higher anxiety for COVID-19 among individuals with adverse early life experiences, e.g., maltreatment. Importantly, childhood maltreatment is also associated with reduced flexibility in appraising the challenges, which mediates the association between maltreatment and anxiety (17).

Another study by LaCaille et al. (18) examined the changes in health behaviors and perceived stress in emerging adults over the first year of college to determine whether pre-pandemic health behaviors were protective of mental health and stress during the initial changes after the COVID-19 pandemic. Sedentary time was found to be increased, whereas physical activity decreased over time. However, 20–35% of students reported improvements in these behaviors. Dietary changes appeared to be mixed, with some improvements noted early during COVID-19. Their perceived stress increased over time. The authors also looked for protective effects on mental health and stress during the pandemic based on the subject’s health behaviors, and diet quality emerged as a significant predictor.

### Psychological Toll and Coping Ways in Elderly Adult Population

The elderly populations were on the receiving end during this COVID-19 pandemic due to the increased risk of mortality and morbidity. This risk has been exacerbated by the high incidence of comorbid conditions found in this population. Consequently, the mental health of the elderly population was affected to considerable degrees during the pandemic. An important survey in 2021 was conducted to determine the effect of stressors on the mental health of people >55 years and found that 32% of the people screened positive for either depression, anxiety, or loneliness (19). Another study by Eastman et al. (20) investigated the relationship between symptoms and changes in alcohol consumption in the same age group and reported that 11% of the people increased their alcohol consumption during the pandemic. Furthermore, this study also found that participants who screened positive for either depression, anxiety, or loneliness had a greater risk of increasing their alcohol consumption during this time. Whitehead (21) found that more calamitous expectations of the pandemic increased perceived stress in its participants (>60 years), which led to an overall negative effect on this population.

Broadly, coping mechanisms/skills also formed an integral part in dealing with the negativities of the COVID-19 pandemic. One major study by Pearman et al. (22) found that proactive coping strategies and resilience helped to lessen the stress in elderly adults compared with younger people.

One of the surveys by Finlay et al. (23) investigated various coping methods used by the participants and reported that positive methods, namely, exercising, adjusting attitudes and modifying pre-set routines, and staying socially connected, as well as methods that were health-limiting, like overeating, were main adaptations by this population. However, another study examined people more than 65 years and categorized coping mechanisms into two types, namely, problem-focused coping and emotion-focused coping (24). The former mechanism entailed taking precautions against getting infected, and the latter one focused on including a daily routine, creative activities, and connecting with other people, which was similar to findings of other survey-based studies. Vannini et al. (25) reported that higher resilience was associated with increased use of adaptive coping and decreased use of maladaptive behaviors. In this study, resilience was found to be the strongest predictor of stress, and the high values of resilience decreased the effects of self-blaming. The authors also found that resiliency training exercises can lead to better preparedness regarding stronger mental health during such situations. Minahan et al. (26) showed that maladaptive coping methods can lead to detrimental outcomes, wherein avoidant coping mechanism was a strong contributor to COVID-19-related stress, depression, anxiety, and loneliness.

Rutherford et al. (27) assessed COVID-19 as a trauma stressor and compared the elderly population with preexisting PTSD who reported that living alone and along with physical illness were associated more frequently in comparison with the control group, i.e., without preexisting PTSD (*p* = 0.02). Contrastingly, Hamm et al. (28) examined the elderly adult population with preexisting depression and found that these were more concerned about the risk of contracting the virus than the risks of isolation. This population exhibited better resilience and had virtual contact with friends and family. However, their quality of life suffered due to continued physical distancing. However, the depression, anxiety, and suicidal ideation symptom scores did not differ as compared with pre-pandemic scores.

### Psychological Toll and Coping Mechanisms in Healthcare Workers

In a survey by Shechter et al. (29) in the New York area (the most severely affected city with COVID-19 in the United States), hospital staff reported depression (48%), anxiety (33%), and stress (48%) in HCWs. Although more than half (61%) reported an increased sense of meaning/purpose since the COVID-19 outbreak. The authors also reported that the most common coping behavior was physical therapy or exercise, while most responders were interested in an individual therapist with online self-guided counseling. Comfort et al. (30) conducted a study on HCWs from an outpatient clinic in April–June 2020 and assessed their mental health challenges during the COVID-19 pandemic. The authors found that there was a two-third increase in stress levels, whereas there was a one-third increase in anxiety/depression levels. The reasons were mostly due to patient care, worrying about contracting and spreading infection, work- and home-related concerns, burnout, and fear of the unknown. Other contributory factors were lack of personal protective equipment, difficulty coping with co-worker illness, and absence (30).

Another study was conducted by Dehon et al. (31) among emergency care physicians (October–December 2020) to assess the psychological effects of the COVID-19 pandemic, and the authors found that predominantly, negative psychological effects were reported. These included feeling more stressed (31%), lonelier (26%), more anxious (25%), more irritable (24%), and sadness (17.5%), respectively. The prevalent psychiatric conditions were insomnia (18%) followed by depression (17%), anxiety (13%), and PTSD (7.5%), and these were prevalent mental health conditions. The major coping mechanism used by this population was avoidance of coping strategies like denial, substance use, venting, behavioral disengagement, self-distraction, and self-blame. These coping skills were among the strongest predictors of psychological distress, whereas humor and positive reframing were negatively associated with psychological distress among these subjects. Feingold et al. (32) assessed for posttraumatic stress growth during the second wave of COVID-19; HCWs in an NYC hospital reported greater appreciation of life, improved relationships, and personal strength when compared with the baseline characteristics during the first wave, which was around 7 months earlier.

In addition, Zoorob et al. (33) conducted a study in April 2020 among resident physicians and found that being male and aged above 39 years were associated with favorable wellbeing indices (*p* < 0.01), and they reported that the institutional support was a favorable contributor in this regard. However, various mindfulness practices were not found statistically significant for the improvement of wellness or resilience factors by this study.

Czeisler et al. (34) conducted a study during the COVID-19 pandemic to identify factors associated with adverse mental health symptoms, substance use, suicidal ideation, and the prevalence among unpaid caregivers of adults versus non-caregivers and reported that caregivers had significant and higher prevalence rates than non-caregivers in terms of adverse mental health symptoms including suicidal ideation (33.4 vs. 3.7%; *p* < 0.0001). The authors identified that the younger age group caregivers were disproportionately affected and found the urgency to access for mental healthcare resources to address their mental health challenges, especially among caregiving individuals.

### Psychological Toll and Coping Ways in Pregnant and Postpartum Women

Typically, women experienced added difficulties compared with the general population during the COVID-19 pandemic, especially those who were in their peripartum period. This was due to decreased opportunities for routine obstetric care and available beds in hospitals. Barbosa-Leiker et al. (35) conducted an important study on peripartum women (*n* = 162) to examine stressors and types of resources required by them during the COVID-19 pandemic. The authors reported that 31% of pregnant participants vs. 5% postpartum had missed their appointments (*p* = 0.007), consequently leading to an increase in stress and anxiety levels, and this reason declined their nutrition levels. This increase in anxiety was attributed to fear of the fetus contracting COVID-19 infection (52% pregnant and 49% postpartum), followed by self or partner contracting the virus (38% pregnant and 32% postpartum), respectively. In addition, the authors reported that 41% of pregnant women and 19% of postpartum women used telemedicine to continue their routine obstetric care and found satisfaction with this new telemedicine process; 32% of postpartum women also revealed less access to cleanliness resources and baby supplies. The most prevalent reason for stress in 21% of pregnant women was a financial decline in income (35).

Another large survey was conducted by Kinser et al. (36) in peripartum women (*n* = 527) to investigate the predictors of depression, anxiety, and PTSD. The authors found that most reasons were related to job insecurity, concerns about family, comfort foods, resilience/adaptability score, sleep, and use of social and news/media.

The study findings further added that social media had played a key role during this pandemic time. However, it was reported that continuous use of social media leads to “pandemic fatigue.” Moreover, 67% of pregnant and 73% of postpartum women reported that “taking a break from watching, reading, or listening to news stories, including social media” was one of the predominant methods of coping (35). In contrast, another study by Kinser et al. (36) reported that 48% of women reported an increase in the duration of social media use, and it was found that this was significantly associated with depressive symptoms (*p* = 0.013) and PTSD symptoms (0.002). However, various women also commented on the methods for self-care from this, and it was noticed that a principal theme was to avoid news and social media completely due to reasons that anxiety arising due to these can further add up to the normal stresses of childbirth and childcare, whereas another group of women suggested a different approach to social media, wherein they used it to connect personally with friends and family, which helped them to build social support and a sense of comfort. Therefore, making social media use and acting on decreasing the “sensational content” makes it a productive rather than detrimental tool which is similar to the use in the general population. Other coping mechanisms included exercise, healthy diet eating, mindfulness practices, connecting with loved ones, and getting good sleep (35). The authors also examined the disparities and factors associated with economic issues and reported that women with higher income were able to engage more regularly to take care of themselves (*p* = 0.007) and connect with other persons (*p* = 0.047) in comparison to women with lower income status who had financial problems (*p* = 0.03) (35). This typically reflects on the disparities contributed by a socioeconomic divide within the population, where persons from the lower end of strata not only have less access to healthcare but also face challenges in their personal life coping physically and emotionally with the burdens of the pandemic.

Finally, this pandemic became more devastating for the peripartum women who already exhibited depressive symptoms during the pre-COVID-19 period. Anderson et al. reported in a study that around 75% of the population had worsened their mental health, with 31.7% of women having clinically elevated depressive symptoms and 36.7% for anxiety symptoms. These subjects also reported a re-emergence of mental health symptoms. Self-isolation was found to be associated with depression, whereas spending time outdoors was found to be negatively associated (37). The authors also showed the role of wellness behaviors in decreasing psychological effects during a pandemic for at-risk population groups.

### Psychological Toll and Coping Ways in Minority Groups

Various races/communities, especially African American people, were affected disproportionately by this COVID-19 pandemic, which remained a controversy due to the sociocultural divide. A community-based survey was done in April 2020 by Saltzman et al. (38) to assess this among 341 participants, and it reported that those people who identified themselves as Latinx, Latina, and Hispanic had a 10 times more probability of having a threshold score for depression (38). Another survey by Ornelas et al. (39) was conducted to determine “how these Latina immigrants residing in King’s County coped with this pandemic” and reported that only a minority of women got infected with COVID-19; however, only 23% of them underwent the COVID-19 testing. This study also found that only 14% reported no knowledge of where to go, whereas 15% were concerned about the cost of the test and 12% did not want to know if they were infected at all. A total of 75% of the participants were worried about the housing and food costs. All these factors lead to a moderate range of depressive and anxious symptoms (38).

Another detailed survey was conducted by Garcini et al. in a Latinx community and reported six different themes of stressors, i.e., economics, immigration, misinformation, family-related concerns, healthcare, and social isolation. This community especially coped with COVID-19 *via* changing their behavior, collective thinking as a community, and spirituality (40).

Mainly, African American community was also affected directly and indirectly by this COVID-19 pandemic. A prospective study by DeSouza et al. examined the effects of the closure of African American Churches (popularly known as “Black Church”) that led to increased mental stress in the followers/believers. The reason being that these Churches served as a historical and a cultural symbol for them and these places helped to improve their mental health as a community to deal with racism (41).

Parker et al. investigated the African American Adolescent’s population perceptions of their experiences and reported that most of the participants had difficulty adjusting to their changed routines including virtual learning and simultaneously had to deal with great anxiety. These adolescents coped with this pandemic using problem-solving methods and getting social support from families and school members, even spirituality also played a part in coping strategies (42).

Another survey was done by Choi et al. on the Korean immigrant (population *n* = 790; the United States and Foreign-born) and found increased levels of stress during the COVID-19 pandemic. The authors predicted various critical factors for this such as a person’s resilience, experiences of day-to-day discrimination, and perception of racial discrimination toward Asians. This study stressed how superficial perceptual tendencies and bias toward a single racial group can be detrimental factors to the mental health of the community and advocated that various measures should be implemented to educate the general population about the risks as well as the redundancy of such behavior (43).

Another study by Pirutinsky et al. was conducted on the American Orthodox Jews population (*n* = 419) and reported high levels of concern and compliances with COVID-19 guidelines and lower stress levels. These were associated with higher religiosity, religious coping, and trust in a higher power leading to lower stress. This study shows that in a close-knit religious community, the faith-directed coping strategy could promote a higher resilience during times of crisis (44).

Overall, these studies show that communities of color experienced a disproportionately increased stress during this COVID-19 pandemic too. However, it is attributable to the health care disparities in this population (leading to high mortality and morbidity within these groups). Authors also found that racism, racial bias and hatred, financial constraints, and lack of information also contributed to the declining mental health during the pandemic in these groups. Authors stressed that a common coping strategy among this population was faith and religion, with communities rallying around their beliefs and social support to form a cohesive unit to tide them through this time of crisis.

## Discussion

Most of the surveys included a coronavirus anxiety scale (CAS), which is a 5-item screening tool and was developed for clinical research and practice. This scale has good reliability and validity measures (90% sensitivity and 85% specificity) (45). Overall, data by Understanding America Study (UAS) suggests a substantial increase in psychological distress during the early COVID-19 pandemic period in March 2020 as compared with June 2020 based on PHQ-9 tools. This decline suggested induction of resilience at the population level (46).

The above extensive rapid review of literature validates the huge burden on mental health associated with the COVID-19 pandemic. The impact varied across different population groups and various coping mechanisms evolved. As the studies in different groups were noted, the authors identify a framework for the increased burden of mental health illness. The reasons can be broadly classified into three categories: (1) Individual factors, (2) Environmental factors, and (3) Disease-related factors.

(1) Individual factors include baseline mental health or mental illness even in the pre-pandemic phase that is now exacerbated due to several factors including deprived social interaction or loss of family member during the pandemic, limited access to psychiatric or psychological care, and substance use disorders.

(2) Environmental factors include the financial stress experienced with illness, hospitalization, accessing healthcare through COVID-19 illness, employment status, impact on several businesses, increased family time at home, the impact of social media, political controversies related to vaccination, and management of illness across times.

(3) Disease-related factors include the severity of COVID-19 illness, isolation associated with illness, and worsening co-morbid medical conditions in the context of the COVID-19 pandemic.

The etiology of mental illness is multifactorial and heterogeneous. The multifactorial nature of mental illnesses would incorporate various components like individual factors, environmental factors, and disease-related factors. Accordingly, the coping strategies need to be tailored based on the individual etiologies. Overall, the health system needs to identify these important public health concerns and optimize its resources including increasing the primary care provider screening and public resources to tackle and mitigate the increased mental health illness associated with the COVID-19 pandemic. The importance of characterization of these risk factors from a clinician’s perspective would be important as this can guide the suggestions on coping mechanisms or explore support resources in alignment with the respective etiology.

Some risk factors are uniquely positioned in that they can be the etiology in some, while others might be the coping methods in other groups. For example, working from home which is an environmental factor resulted in satisfaction for many employees as it increased family time and flexibility in daily life. Unfortunately, the performance pressure related to working from home increased stress, and also more family time was associated with some family discordance in some individuals. Many families separated by geography and limited by travel to connect utilized technology to connect and bridge, but unfortunately, this could not fulfill the emotional needs of in-person interactions.

This narrative review highlights psychological issues and coping methods and is summarized in Table 1. To note is that mental illness is an underreported and underdiagnosed problem in the community and without a doubt is also hugely under-identified amid the COVID-19 pandemic, which primarily presented as a medical illness but had a huge mental health impact.

Public resources to tackle mental health illnesses associated with this pandemic need a special focus. More resources need to be allocated and access to psychiatric care and psychological health evaluation needs to be increased. Primary care providers need to step in as a bridge to screen for mental health illnesses and refer to appropriate resources and follow up promptly. The end of the COVID-19 pandemic should not be the origin of a new mental illness pandemic, and as a society, we need to address this timely and mitigate it.

### Strengths and Limitations

This rapid review has synthesized the evidence and discussed the currently available literature on the psychological challenges and coping ways during the COVID-19 pandemic on all the age groups, especially vulnerable groups like children, elderly, women, and HCWs. To our knowledge, this is the first rapid review investigating the United States population groups who were vulnerable in the context of psychological challenges, coping ways, and public support. However, this pandemic has affected almost every country on the earth and disrupted everyone’s living in a way that no other outbreak has in our memory. The major strength of our review is that it aspired toward greater inclusion in a rapidly changing COVID-19 landscape, while we adhered to a standard methodological approach and assessed the study quality and risk of bias using the GRADE approach. We followed best practice principles to evaluate the certainty of evidence, and we presented a tabulated and narrative synthesis. Our review has clear limitations in several forms as the majority of the studies included in this review were from the United States. Our major limitation of the review was that no empirical studies investigated this impact on these vulnerable populations, and thus, there is limiting generalizability to the general population. Our inclusion criteria did not include studies from any other developed countries and studies in languages other than English, limiting the generalizability of our results. We were not able to register on PROSPERO, and only two reviewers were responsible for the initial screening of papers and quality assessments.

Finally, our review’s searches were carried out later in the pandemic, and it will be considered to match the emerging research from the other countries over the globe in the light of our review’s findings.

## Conclusion

This rapid review confirms that children, pregnant women, the elderly population, and HCWs were more at risk of significant psychological challenges, and they have developed various coping ways to show resilience during the COVID-19 pandemic. Various published survey studies suggest that symptoms of depression, distress, and anxiety are commonly found within these populations and lead to a significant impact on Mental Illness, Culture, and Society. We recommend more research be undertaken to identify interventions and personalized psychological approaches that can be delivered to mitigate the deterioration of people’s wellbeing and support their mental health.

## Author Contributions

RK, SP, AS, JB, RM, and US contributed to the conception and design of the study. RM and AS organized the database. US performed the statistical analysis. RK wrote the first draft of the manuscript. SP, AS, RM, JB, and US wrote the sections of the manuscript. All authors contributed to manuscript revision, read, and approved the submitted version.

## Conflict of Interest

The authors declare that the research was conducted in the absence of any commercial or financial relationships that could be construed as a potential conflict of interest.

## Publisher’s Note

All claims expressed in this article are solely those of the authors and do not necessarily represent those of their affiliated organizations, or those of the publisher, the editors and the reviewers. Any product that may be evaluated in this article, or claim that may be made by its manufacturer, is not guaranteed or endorsed by the publisher.
